# Biomedical publishing: Past historic, present continuous, future conditional

**DOI:** 10.1371/journal.pbio.3002234

**Published:** 2023-10-03

**Authors:** Richard Sever

**Affiliations:** Cold Spring Harbor Laboratory, Cold Spring Harbor, New York, United States of America

## Abstract

Academic journals have been publishing the results of biomedical research for more than 350 years. Reviewing their history reveals that the ways in which journals vet submissions have changed over time, culminating in the relatively recent appearance of the current peer-review process. Journal brand and Impact Factor have meanwhile become quality proxies that are widely used to filter articles and evaluate scientists in a hypercompetitive prestige economy. The Web created the potential for a more decoupled publishing system in which articles are initially disseminated by preprint servers and then undergo evaluation elsewhere. To build this future, we must first understand the roles journals currently play and consider what types of content screening and review are necessary and for which papers. A new, open ecosystem involving preprint servers, journals, independent content-vetting initiatives, and curation services could provide more multidimensional signals for papers and avoid the current conflation of trust, quality, and impact. Academia should strive to avoid the alternative scenario, however, in which stratified publisher silos lock in submissions and simply perpetuate this conflation.

This article is part of the *PLOS Biology* 20th Anniversary Collection

## Introduction

Communicating the results and conclusions drawn from research is essential for scientific progress. Scientists have a duty to share reports of their work so that the community can critique and build on it, extend human knowledge, and ultimately translate the findings into tools that benefit society. Journals have played a key role in selection and distribution of research reports in the biomedical sciences for more than 350 years [1]. Science publishing has become a multibillion-dollar industry that peer reviews and publishes millions of research articles each year. These articles are critical not only for communication between scientists but also for them to demonstrate their productivity and thereby secure grants, jobs, and promotions in a hypercompetitive academic environment.

Here, I review the history of science journals, discussing how peer review first emerged and later became widespread in the late 20th century, the rise of the journal brand, and how publishers capitalized and adapted journals to the online world. I then describe how journals currently operate and their roles in verifying, peer reviewing, and filtering submissions, before discussing how publishing could be improved in the era of the Web. My focus is primarily on when and how articles are shared and evaluated. I touch on some of the technical and financial aspects, but a comprehensive treatment of publishing infrastructure and business models is not my aim here. For the most part, I draw examples from biomedical science but many of the concepts discussed apply more broadly across academic publishing.

Briefly, I argue that we are at a point where we can take full advantage of the potential of the Web for science communication. It is time to rethink the extent to which a single entity should be responsible for disseminating and evaluating research. Instead we should build a more decoupled system in which articles are first disseminated as preprints and then undergo review, filtering, and evaluation once they are already publicly available. Addiction to journal brand and Impact Factor has led us to conflate quality, impact, and trust in research. We now have the opportunity to adopt a new approach with better trust signals, in which judgments of an article’s merits are not made at a single point in time. However, we are at a fork in the road where we can choose a more open and equitable path or one that locks articles into publisher silos.

## The past historic

**Fig 1:**
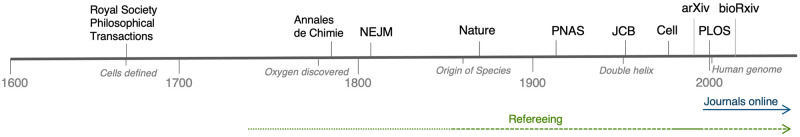
A timeline of science publishing. The timeline shows several milestones in the history of science publishing, including the launch of various journals. It is not intended to be comprehensive. The focus is on publications in the field of *PLOS Biology* but some titles in other fields are also included. Not shown for the sake of clarity are the launch of *Nature Genetics* (1992, *Nature*’s first successful spin-off journal), BioMed Central (2000, first Open Access publisher), *F1000R* (2013, first post-publication peer review), and *PLOS ONE* (2004, first mega journal), which should also be considered milestones. The timeline of implementation of external peer review is also indicated. Refereeing was introduced in the early 18th century but did not become standard until the late 20th century. The launch of preprint servers (e.g., arXiv and bioRxiv) meant scientific articles were again frequently available without formal peer review. In the figure, a dotted line indicates refereeing existed, a dashed line indicates when it became more common, and a solid line indicates when it was near universal for research dissemination.

A timeline of some key milestones in biomedical publishing is shown in Fig 1. Up until the 17th century, scientific discoveries were communicated primarily in private letters and monographs. The first scientific journal—*Philosophical Transactions of the Royal Society* [2]—was launched in 1665 by Henry Oldenburg (*Journal Des Sçavans* was launched earlier the same year but was broader in scope). Numerous, often short-lived, publications appeared over the next 2 centuries, but it was in the 19th century that journals familiar to readers today began to appear, including *Nature*, *Science*, *New England Journal of Medicine*, *The Lancet*, and the *British Medical Journal* (*BMJ*). Many of the early periodicals were operated by scientific societies. Despite Oldenburg’s initial aspirations, they were generally not lucrative ventures and were often subsidized by the societies that ran them [3].

The mid-20th century saw a rapid increase in the number of journals, reflecting the massive expansion in scientific research around World War II (WW2) and, increasingly, commercial motives. Subscription revenue and, later, author fees proved a highly profitable business model. Publishers like Robert Maxwell’s Pergamon Press (later bought by Elsevier) capitalized by cultivating academics in different subfields as editors and launching numerous new titles across the life sciences [4]. In 1850, there were approximately 1,400 journals; come the 21st century the number had risen to more than 20,000 [5]. Journals continued to proliferate into the early 21st century, with a series of mergers and acquisitions among the big commercial publishers resulting in a “big five”—Springer Nature, Elsevier, John Wiley, Sage, and Taylor & Francis—controlling most of the industry.

### Peer review revisionism

Early journals naturally needed editors like Oldenburg to solicit and select manuscripts. The use of peer reviewers is sometimes wrongly assumed also to be a centuries-old standard that existed from the outset. In fact, the practice of consulting external referees was only introduced in the 18th century and it was not universally adopted until much later. Moreover, where external referees were used, the aim was generally to protect the reputation of the scientific society that published the journal rather than to assess the merits of articles [1,6].

Peer review did not become standard until the latter part of the 20th century. Many editors continued to handle the vetting process alone until after WW2. For example, whereas a number of learned societies began to send submissions to external referees in the late 19th century, *Science* and the *Journal of the American Medical Association* (*JAMA*) did not routinely do so until after the 1950s and *Nature* and *The Lancet* waited until the 1970s [1,7–9]. Part of the reason for bringing in external referees was the increasing volume of highly specialized submissions editors had to evaluate, but pressure for greater scientific accountability given the huge increase in government spending appears to have been a key driver. This was also the point when the term “peer reviewer” started to replace “referee,” reflecting a desire that decisions about science be made by experts to avoid political interference [7].

As peer review evolved, it began to serve a role beyond simply helping editors filter out inappropriate articles: Referees were also asked to assess the quality of the research. This came to encompass evaluation of both its soundness and its significance, a combination that remains controversial. Judgments about the significance of the research became increasingly important commercially as journals competed for authors and subscribers, while funders, readers, and employers sought quality filters in the face of a growing deluge of literature. The perceived prestige of a journal became a key variable to which referees began to calibrate their reviews.

### Brand new world

Competition among journals led to the emergence of formal and folk rankings of titles. This helped librarians with limited budgets make subscription choices; more problematically, it provided a quick and easy proxy for the quality of individual articles in those journals. Eugene Garfield at the Institute for Scientific Information (later part of Thomson, then Clarivate) developed the most widely used metric—the Impact Factor—in the 1970s [10]. A ratio that approximates the mean number of citations to qualifying articles from a two-year period, the Impact Factor is a particularly inappropriate measure of the quality of any single article given the skewed distribution of citations in each journal [11]. Nevertheless, it became an obsession within the academic community, largely because of the often-correct belief that peers, funders, and institutions used Impact Factors to assess individual scientists. “Impact Factor” remains the most common co-search term in Google for almost any journal, and editors frequently note it is the first thing prospective authors ask about.

**Fig 2:**
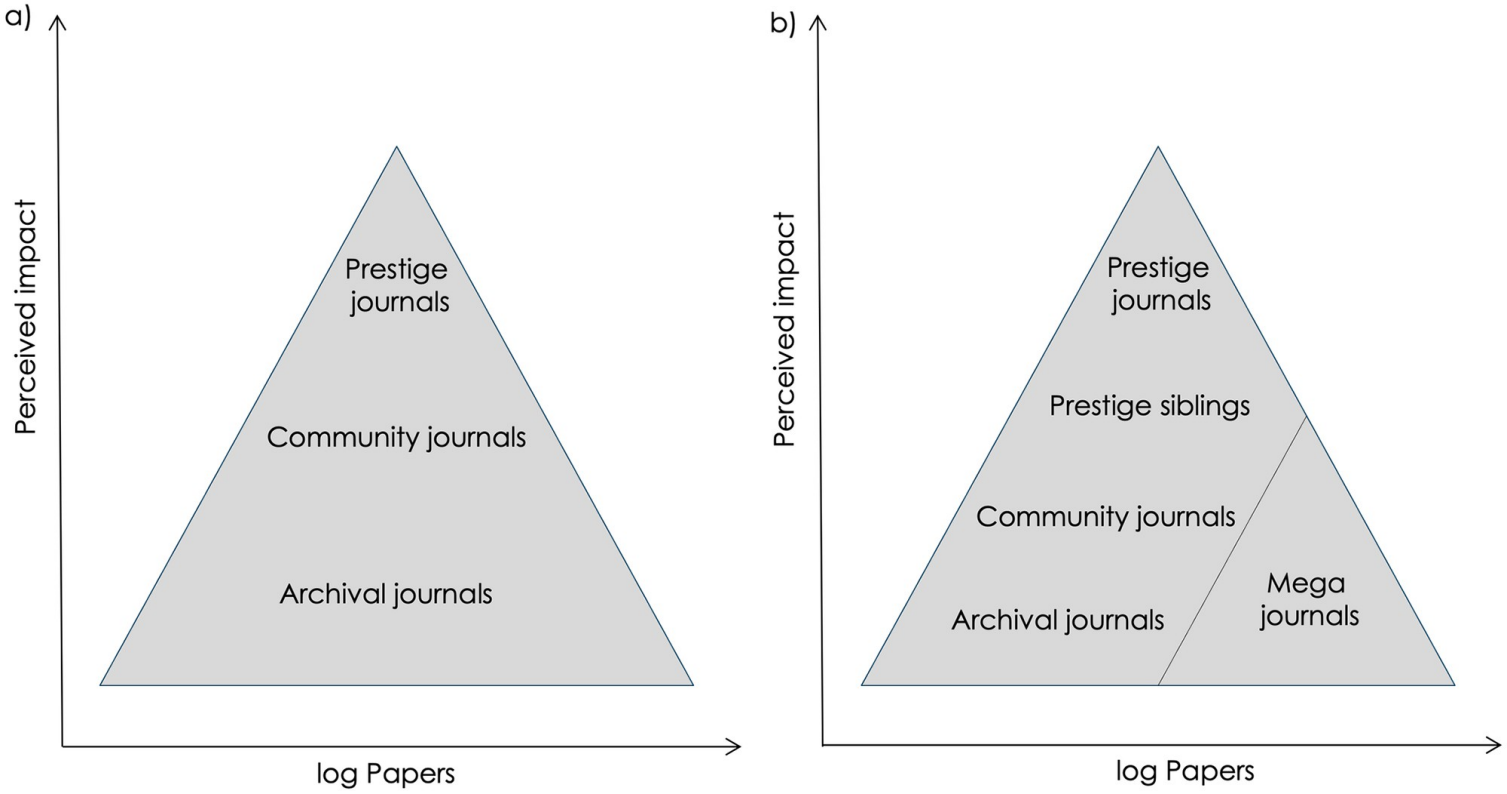
Journal hierarchies. Most biologists select the journal to which they submit based on their judgment of where a paper fits in a hierarchy of journal impact that typically correlates with Impact Factor. Significant changes in the past 2 decades have been the arrival of sibling journals of prestige titles (**b**) that have in effect relegated community journals that previously occupied the second tier of the pyramid (**a**) and mega journals.

Perception of a journal’s quality has of course never been solely about metrics. Many titles developed reputations for the fairness and diligence of their evaluations, together with authority conveyed by the academics on their editorial boards. Thus as academics began to view journals as a hierarchy (Fig 2), this generally correlated with Impact Factor but not always; a strong brand could act as an indicator of quality independently or even in the absence of an Impact Factor (this has been particularly important for new journals, given qualification for indexing and award of an Impact Factor can take years).

Publishers were highly effective at capitalizing on journal brands, ultimately cashing in on prestige titles by launching sibling journals. *Nature* first attempted this in 1971 with the short-lived *Nature New Biology*, edited by Ben Lewin, and *Nature Physical Sciences*. Ironically, these were soon shuttered amid financial concerns and worries about dilution of the brand. There would be no such concerns 20 years later after *Nature Genetics* was launched. Lewin meanwhile launched *Cell* in 1974, cultivating a reputation for publishing groundbreaking science—and only science Lewin believed was groundbreaking. A publication in *Cell* became something to crow about and soon where one published seemed to matter as much as what one published. This was a key step in the rise of journal brands, to which the scientific community became as addicted as they were to Impact Factors. As *Nature* later budded off *Nature Genetics*, *Nature Medicine*, and *Nature Structure*, *Cell* too spawned siblings like *Neuron* and *Immunity* then *Molecular Cell*, *Developmental Cell*, *Cancer Cell*, etc. Other publishers would create similarly branded portfolios, including *The Lancet*, *JAMA* (by rebranding existing titles), and *BMJ*. Sibling journals reinforced the parent brand, provided an outlet for rejected papers and diverted submissions away from the journals that previously occupied the second tier in the journal hierarchy (Fig 2b). They also represented a key tool for retaining authors within a publisher’s stable as online publishing provided an additional stimulus to launch “trickle-down” journals that accepted papers rejected by the parent title.

### Fine margins online

The arrival of the Web led to major changes in science publishing. Publishers had moved to digital composition by the 1980s but only started publishing articles online in the late 1990s. As online publishing matured, articles were augmented with increasingly reader-friendly HTML, high-resolution images, movies, and other supplementary material. XML was introduced as the indexing/archiving format, and the online article became the version of record. Meanwhile, authors had to switch from mailing in manuscripts to uploading files via dedicated online submission systems. Of only a handful of widely used submission systems, Elsevier acquired Editorial Manager, John Wiley acquired eJournal Press, and Clarivate acquired Manuscript Central. Market consolidation is another theme here.

Importantly, the Web also altered the economics of publishing. An in-depth discussion of its impact is beyond the scope of this article, but a few important effects are worth noting. Publishers adapted the subscription model to the Web very effectively, creating so-called “big deals” or other packages that offered institutional subscribers bundles of titles. This further strengthened the hands of big publishers. New journals could easily be added to existing deals. Meanwhile, automated production processes generated economies of scale that also benefited big publishers and increasingly allowed them to attract scientific societies and other smaller publishing operations as clients.

Another consequence was the birth of Open Access (OA). Calls to take advantage of the Web to make articles freely available to everyone led to a debate about whether this should be accomplished by simply archiving free copies of manuscripts published in subscription journals (so-called “Green” OA) or by switching to a model in which article-processing charges (APCs) levied on authors or funders covered the cost of publication in a new breed of “Gold” OA journals that had no paywalls. Gold OA, increasingly seen as the desired solution by some funders and commercial publishers [12,13], had an important knock-on effect: it incentivized publishing more papers, since every accepted paper could be monetized via an APC (for subscription journals, by contrast, publishing additional papers incurs extra costs without providing additional revenue). This in some respects provided a counterforce to the endless pursuit of Impact Factor and led to the launch of mega journals such as *PLOS ONE* and *Scientific Reports* that selected only for “sound science” rather than significance, which many hoped would encourage people to judge papers based on their own merits (or at least their own metrics) rather than the journal in which they were published. A new generation of born-OA commercial publishers, including Hindawi, MDPI, and Frontiers, also capitalized on the approach, generating remarkable growth in recent years but also concerns about the integrity of their review processes [14]. Many traditional publishers, meanwhile, launched less selective Gold OA siblings alongside existing titles to generate new revenue streams and hedge against the loss of income anticipated in a move away from subscriptions.

Changes in the ways researchers discovered papers online provided an additional potential push to article-based evaluation. Web-based tools like PubMed and Google Scholar replaced paper- and CD-ROM-based indexes and also reduced the kind of general browsing readers had performed with paper copies. Online search results centered individual articles rather than journals by increasingly making articles, not tables of contents or journal home pages, the literature-entry point. However, the fact that submissions to mega journals soon appeared to track their Impact Factors indicated the Impact Factor addiction persisted [15]. Meanwhile, journal brands labeled individual articles just as effectively as entire journals, and Web domain names only served to reinforce brand identity.

### Preprints: Prehistory repeating

Perhaps the greatest threat to publisher hegemony in the online world was increased use of preprints, manuscripts that have not (yet) been peer reviewed by a journal so lack any brand imprimatur. Drafts of manuscripts had always been shared among small groups of academics, but the Web meant this could occur globally. The physics community was early to take advantage with the arXiv server, set up in 1991 [16]. There were a number of attempts to replicate the practice in biomedical sciences, including a proposal for an NIH initiative [17], but it only gained traction in 2013 with the launch of the preprint server bioRxiv [18]. Preprint servers speed up dissemination by allowing readers to access manuscripts immediately rather than after the significant delay caused by peer review and, in addition, have provided a simple, low-cost way to achieve OA [19].

While some have raised concerns about dissemination of new scientific results without peer review, it’s important to note servers like arXiv, bioRxiv, chemRxiv, and medRxiv do vet submissions for inappropriate material [16,18,20]. Their screening processes thus in some respects resemble the kind of filtering scientific journals performed for the first few hundred years of their history. The fact that >70% of preprints go on to be published in peer-reviewed journals [16,21,22] and that there are typically only minor differences between the preprint and the journal version [23,24,25] should allay fears they preclude subsequent journal peer review or lead to a significant deterioration in the quality of scientific evidence.

By covering the costs of dissemination and decoupling it from peer review, preprint servers provide a platform for evolution of publishing. This has prompted many stakeholders to rethink the roles of journals, how and when peer review should occur, and perhaps whether it should happen at all. Before discussing the kind of system that could emerge as a consequence, however, it’s important first to understand how journals currently operate and the different roles they perform.

## The present continuous

**Fig 3:**
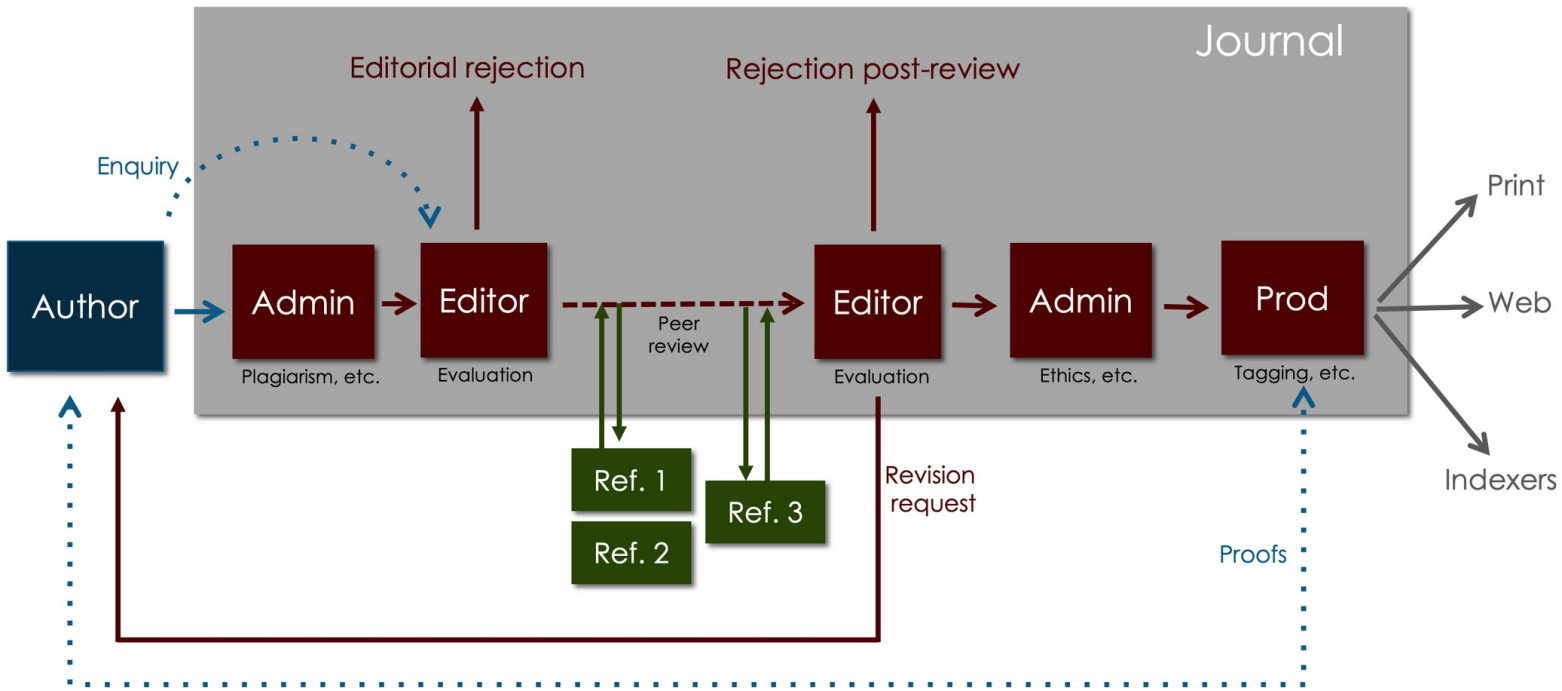
The traditional journal publication process. Authors submit to journals via online platforms, sometimes after a presubmission enquiry, and the journal assigns an editor to evaluate the paper after a series of administrative checks. The editor then decides whether to review the paper, sending papers deemed worthy to experts in the field for assessment (referees). If conflicting reports are obtained, the editor may seek additional referees. Based on the reviews, the editor decides whether the paper should be rejected, accepted, or returned for revision. Revised submissions may be sent back to the referees. Once a paper is accepted, it undergoes further checks before being processed for online and in some cases print publication.

Journals play various roles. Their formal functions include editorial evaluation, various forms of content verification, peer review, text and figure editing, typesetting and content tagging, proofing, digital object identifier (DOI) assignment, print and online publication, long-term preservation, and content deposition (Fig 3). Once an article is published, the journal is responsible for updating it with any necessary corrections and investigating any integrity issues that arise. Journals also serve as discovery platforms and provide qualitative and quantitative information about the articles they publish.

Not all journals perform all of these roles, not all do them well, and not all of these roles are necessary or even desirable. Nevertheless, it’s important to acknowledge they exist, understand why and be cognizant of the costs involved (Box 1) if we want to explore how the system could be improved.

**Box 1. The cost of evaluation.** Defining the costs of publication depends on what one means by “publication.” Converting submissions into HTML and XML and typesetting for PDF/print are relatively inexpensive (though not as cheap as often imagined), as is online hosting. Costs mount, however, when people are involved. Quality control in production, administration, and editing involve salaried staff, and a journal that performs rigorous editorial assessment, verifies ethics, and administers peer review will have per-paper costs 2 orders of magnitude higher ($10^3^/paper) than those of a preprint server ($10^1^/paper). We must also be careful to distinguish costs from charges, as APCs may be subsidized by other sources of revenue (e.g., philanthropy, subscriptions, APCs from other titles with lower costs).

Several high-quality nonprofit journals have made their costs per paper public. The *EMBO Journal* and *PNAS* estimate their costs at approximately $6,000 per published research article [67,68]. *eLife* has in the past produced similar numbers [69]. Note that all these journals are nonprofits, so the costs cannot be dismissed as inflated by the large profit margins of commercial publishers. Meanwhile, despite the high APCs levied at *Nature* (>$10 K/article), the true per article cost is much greater (approximately $30 K/article), largely because of the staff time involved [70]. The *EMBO Journal* estimate that >16 hours of staff time are required per published paper for editorial assessment, content checks and administration alone, and that number would be higher were production tasks included [71]. Note that significant staff time is also spent on articles that are not published and whose costs, at least under a Gold OA model, must be borne by the smaller number of papers that are accepted.

One can argue whether all these costs are necessary and/or whether some could be reduced by AI, etc., but we should not pretend they do not exist. Any future vision for science publishing must either accommodate them somehow or justify why they are not necessary. *eLife* provides an interesting example of one such strategy: by no longer distinguishing articles as accepted or rejected post-peer review—essentially redefining the journal as a peer review service [27]—total costs can be spread over a much larger number of articles, as well as potentially eliminating redundant rounds of peer review elsewhere.

### Editorial review

Authors submit manuscripts to journals either unprompted or after being solicited by an editor. Prior to the Web, solicitations tended to be a feature of selective journals, whose editors actively scouted for interesting research at conferences and lab visits. Solicitations became more widespread with the arrival of email and business models that rewarded high volume—to the extent that academics are now bombarded with spam invitations from a range of journals. Again brand is key: A solicitation from *Nature* will typically be viewed very differently from one sent by an unknown journal, and there is clearly a distinction between a personalized invitation from an editor familiar with the work and a form letter generated using email addresses scraped from the Web.

Once a paper is submitted, the first step is an editorial decision as to whether a paper should be peer reviewed or rejected without external review (desk rejection). The editor(s) will reject submissions that are out of scope or report findings felt to be of insufficient interest or obviously flawed. This is possibly the most controversial step of the process. Selective journals can desk reject >50% of submissions and the percentage is even greater for “high-impact” journals. The fact that decisions at the latter are generally made by professional editors not academics has been a talking point for years. I shan’t reprise the endless arguments but just note the degree of ire correlates with Impact Factor, so this seems more about the stakes than about who is making the decision [26]. It’s no coincidence much of the debate around *eLife*’s decision to abandon post-review accept/reject distinctions [27] focused on the fact papers could still be desk rejected [28]. Regardless of who makes the decision, this is a critical and subjective triage step that occurs very early in the life of a paper and is a key step to consider when re-envisaging science publishing.

### Peer review

For papers that are selected for peer review, in the ideal scenario a journal works hard to find 2 to 3 well-qualified reviewers whose specific areas of expertise, biases, and potential conflicts of interest are known. These reviewers each devote several hours to providing a detailed critique of the paper that gives suggestions for potential improvement, identifies any flaws, and assesses the importance of the advance for the field—there is an ongoing debate as to whether the reviewers should opine on the appropriateness of the work for the journal or leave this to the editors. An editor familiar with the field then thoroughly examines the reports obtained in the context of their own reading of the paper, bringing in additional reviewers if necessary and carefully analyzing any points of disagreement before coming to a decision on whether the paper should be rejected, accepted, or returned for revision. Minor revisions typically involve some rewriting of the text, with acceptance being the expected outcome. Major revisions are more extensive, may require additional experiments, and acceptance is not assured. In these cases, revised submissions will often be sent back to the reviewers for an opinion. Consultative review processes in which the reviewers see each other’s reports before reaching a consensus have given authors further confidence in the process [29], though of course there is a danger of groupthink. The approach may also limit the much-lamented tendency for reviewers to demand major revisions with numerous additional experiments that further delay publication of the work.

A big question is how often that ideal scenario applies. It is increasingly difficult for many journals to obtain reviewers; months can go by as dozens of individuals are invited and decline or just fail to reply. Desperate editors may simply use individuals suggested by the author or automated/algorithmic selections and ultimately settle for anyone who seems vaguely qualified. Meanwhile, the inevitable tendency of editors to rely on people they know skews the reviewer pool to senior academics in Europe and the United States [30]. Conflicting reports are a challenge for any editor and add further subjectivity and sampling bias. Some editors have the time to thoroughly assess areas where the opinions of the reviewers differ, but there is a suspicion that others simply make decisions based on the majority view among 3 reviewers—a formula hardly likely to satisfy statisticians. In the worst-case scenario, the process is corrupted by peer review rings in which individuals conspire to give each other’s papers an easy ride or create entirely fake reviewers [31]. The existence of predatory journals in which peer review is cursory or nonexistent further underscores the conclusion that peer review cannot be seen as any form of standard at a systemic level, however, well operated by good journals. In other words, given this variability, the mere label of something as “peer reviewed” now has little value.

The problem manifests in part because the review process is largely opaque and uncredited, with little to indicate to a reader how thoroughly a paper has been evaluated. An early attempt to establish some form of certification for journal processes called pre-SCORE was unsuccessful [32], perhaps in part because it was journal not article based. More recently, the Docmaps initiative has developed a machine-readable way to represent the different steps in evaluation [33]. A growing trend towards transparent review in which journals publish reviews and editorial evaluations alongside articles they publish may help [34]. More controversial is “open review,” in which referees’ identities are public. While this could in theory reduce the likelihood of malicious reviews by referees hiding behind anonymity, the significant power imbalances in academia make it likely many reviewers, particularly early career researchers (ECRs), would temper even fair criticism for fear of later reprisals. As a consequence, open review has not been widely adopted.

### Content verification

An important but less widely appreciated area where increased transparency about journal processes would be welcome is in the other evaluations they perform. These include various forms of content verification, such as plagiarism checks, screens for image manipulation, ethics checks, verification of data deposits and author contributions, figure correction, copy editing, and nomenclature standardization. The order of events varies: some take place before editorial/peer review, others only once a manuscript is provisionally accepted. It’s important to stress that these processes are far from fully automated (see, for example, [35]). They require significant administrative time as well as editorial oversight to ensure adherence to relevant guidelines that peer reviewers cannot be expected to perform. This is particularly true for clinical studies, where the International Committee of Medical Journal Editors (ICMJE) has defined standards for clinical reporting [36], including important procedures for protection of research participants—for example, verification of informed consent and removal of identifying information. Indeed, the umbrella organization EQUATOR aggregates more than 500 reporting guidelines for health research [37]. Journal policies based on evolving best practices like the Transparency and Openness Promotion (TOP) guidelines designed to improve research reporting and reproducibility are also increasingly common in basic science journals [38].

Ethical responsibilities extend beyond prepublication checks. Journals have an ongoing duty to update manuscripts with any necessary errata (corrections of publisher errors) and corrigenda (corrections of author errors). They must also conduct investigations and liaise with institutional authorities in circumstances where concerns are raised that might lead to complete retraction of an article. These investigations can take a huge amount of staff time and require significant expertise, underscoring the need for humans in the process and associated costs. We should also consider the fact that as business models shift from reader service (subscriptions) to author service (APCs), there is a financial disincentive to increasingly stringent vetting of content. This has manifested most obviously in the appearance of predatory journals but may also affect choices responsible journals make about costly processes such as manual image screening that do not benefit the customer directly.

### Article production

Another area where there has been pressure to reduce costs is production. Biomedical scientists typically submit papers as Microsoft Word files (a minority use LaTeX). Once accepted, article files are converted to a format that allows typesetting of a final print/PDF version and generation of XML and HTML. Meanwhile, figures are converted, sized, and linked and positioned appropriately for HTML and PDF display. The details of the process vary, and there are conflicting views on the ideal workflow. For our purposes, however, it’s sufficient simply to emphasize that while these processes are increasingly automated, they cannot be entirely automated so long as academics use current authoring tools. Human intervention and quality control is necessary, at least for a subset of papers, which are not always predictable in advance. Costs rise further if articles are copy edited or figures are redrawn, both of which involve several person hours per article. Oversight and vetting of proof corrections require further staff time, though some high-volume journals have eliminated these steps. It is also worth emphasizing that movies, executable code, and other novel online features inevitably take up further editorial and production time.

Once an article is published, the publisher typically sends metadata to indexing services such as PubMed and, having generated a unique DOI, deposits this with a registration agency. Complete articles may also be sent to long-term archiving services such as Portico and repositories such as PubMed Central. In addition, the journal will update its own website and use a variety of feeds, emails, and social media tools to alert potential readers to the existence of the article.

### Community and conflation

Journals play another, broader role within the scientific community: They often serve as forums that help define and nurture particular fields. Active editorial boards engage with the wider community of researchers and journals often collaborate with groups of researchers to agree on standards such as the TOP guidelines (see above) for ethics, methods and data reporting in the areas they cover. Together with the editorial checks described above and confidence in the peer review process operated by editors familiar to the community, this potentially allows journals to function as *trust signals* to readers.

More commonly, however, journal titles are used as *quality proxies*. Many institutions have used journal Impact Factors to evaluate and reward faculty, and despite initiatives like the Declaration on Research Assessment (DORA), many scientists suspect institutions continue to rely on Impact Factors when assessing their staff, just not overtly. Journal brand meanwhile is used as a quality proxy in all sorts of informal evaluations when a rapid and rough assessment of an individual is required—from shortlisting job candidates to selecting reviewers, seminar speakers, or award recipients. They are also used by readers as filters to decide what content to browse and which papers to read. Scientists increasingly limit the number of journals they browse and encounter content elsewhere only via targeted searches or other recommendations (human or algorithmic). But even then, journal title often factors into whether they read a paper and what they think of it.

The result has been an unfortunate conflation of quality, trust, and importance in a single parameter: the journal brand. This is made worse by being based on selection performed at a single point in time by a small number of people, who might not be a representative sample of experts in the field. The challenge is to envision a future for scholarly communication that addresses these problems and ultimately provides a path to better trust signals.

## A future conditional

As I have described above, science publishing is currently based on a late-20th century, pre-Web workflow in which journals are responsible for peer and editorial review, content verification, distribution, and correction, and in addition serve as signals for curation and evaluation. The question we should ask ourselves now is how this might all be best performed in the era of the Web. What should the publishing process look like, who should perform each step, and how can we build an ecosystem that is more equitable and avoids a conflation of trust, quality, and impact that may be distorting science? We are now at a point where the idea of decoupling the different publishing functions currently performed by journals has become feasible [39–42], and this could allow us to address these questions.

### Decoupled dissemination

Decoupling of dissemination from evaluation by sharing articles as preprints is now widely—if not universally—accepted, and the COVID-19 pandemic has shown the benefits of immediate availability of scientific findings to be inarguable. There is a growing number of nonprofit preprint servers modeled on arXiv (bioRxiv, chemRxiv, medRxiv, psyarXiv, socarXiv, etc.) to enable this, and the major corporate publishers all operate services that similarly allow posting of submitted manuscripts before completing journal peer review: Springer Nature acquired ResearchSquare; Elsevier acquired the Social Science and Research Network (SSRN); Taylor and Francis acquired F1000R; and John Wiley acquired Authorea. Meanwhile, the majority of journals now allow posting of preprints prior to formal publication, with hundreds actively partnering with preprint servers to receive submissions—see, for example, the B2J process operated by bioRxiv [18]. So-called “overlay journals” in which articles exist solely on the preprint server and are merely reviewed and highlighted by journals would be a logical extension of this process but are yet to be widely introduced in biomedical sciences (see Box 2; [40,43]). Alternatively, production cost savings may be achieved if downstream journals retrieve XML-tagged files from the preprint server (rather than repeating the tagging process), as *eLife* currently does for articles that previously appeared as preprints on bioRxiv and medRxiv. Publishers that operate their own servers and channel authors to final publication venues they operate have a similar financial incentive to create XML files only once.

**Box 2. A blueprint for overlay journals.** Overlay journals take advantage of the fact that articles have already been permanently archived on a preprint server. The overlay journals need not go to the expense of developing and maintaining a hosting service for the articles itself and instead simply comprises lists of articles that have been selected for the journal with links back to the preprint server where the articles reside. The overlay can therefore comprise a lightweight website, perhaps something as simple as a Wordpress site. The journal *Discrete Analysis* operated by the math community on arXiv is perhaps the archetypal example.

**Fig 4:**
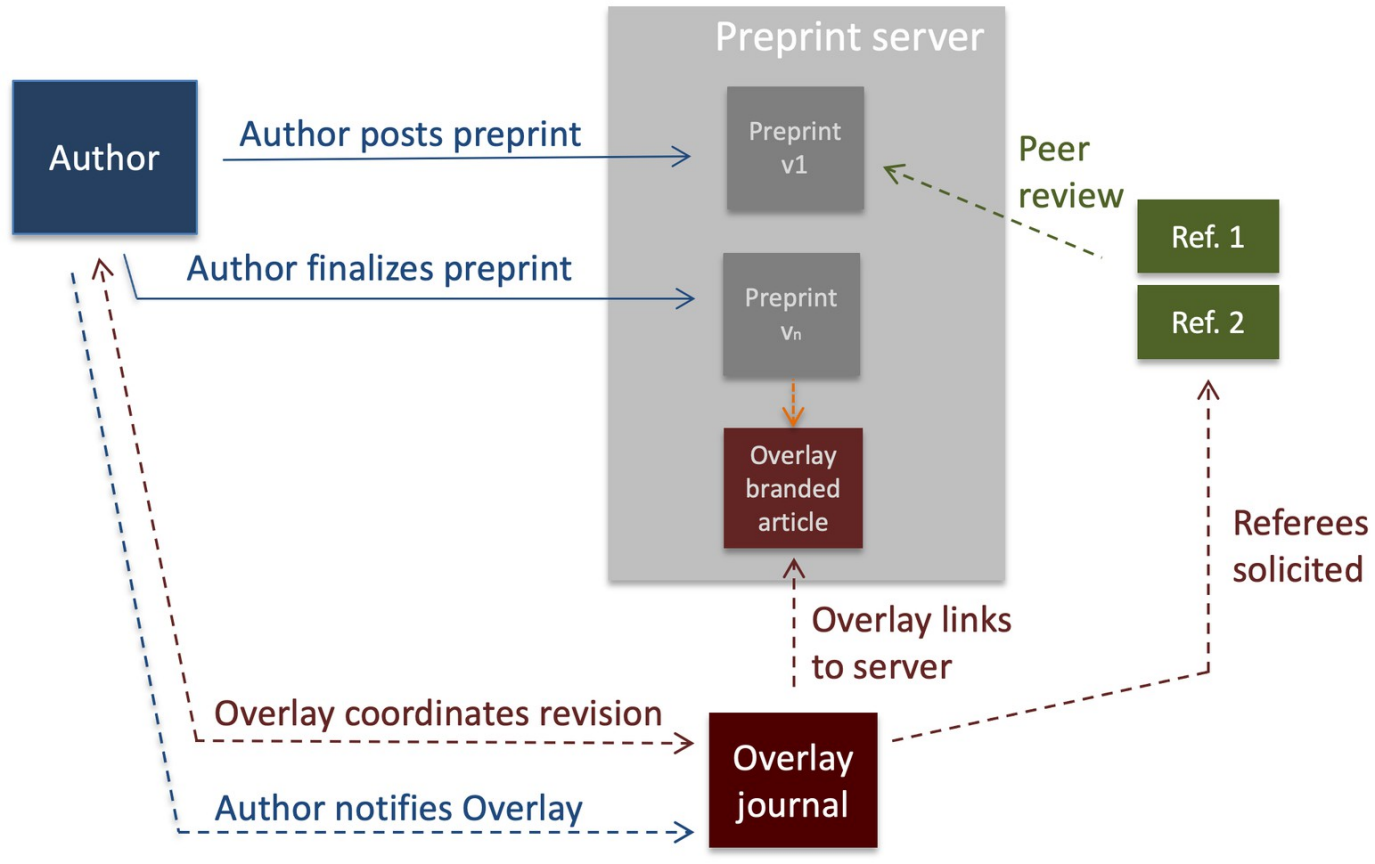
Box 2—Schematic of an overlay journal workflow.

For overlay journals to be successful, however, the final version on the preprint server that has been approved by an overlay journal should be differentiated in some way both to human and machine readers (Fig 4). This could be achieved by display changes at the preprint server, insertion of new metadata, and updating of the DOI record and/or assignment of a new DOI.

**Fig 5:**
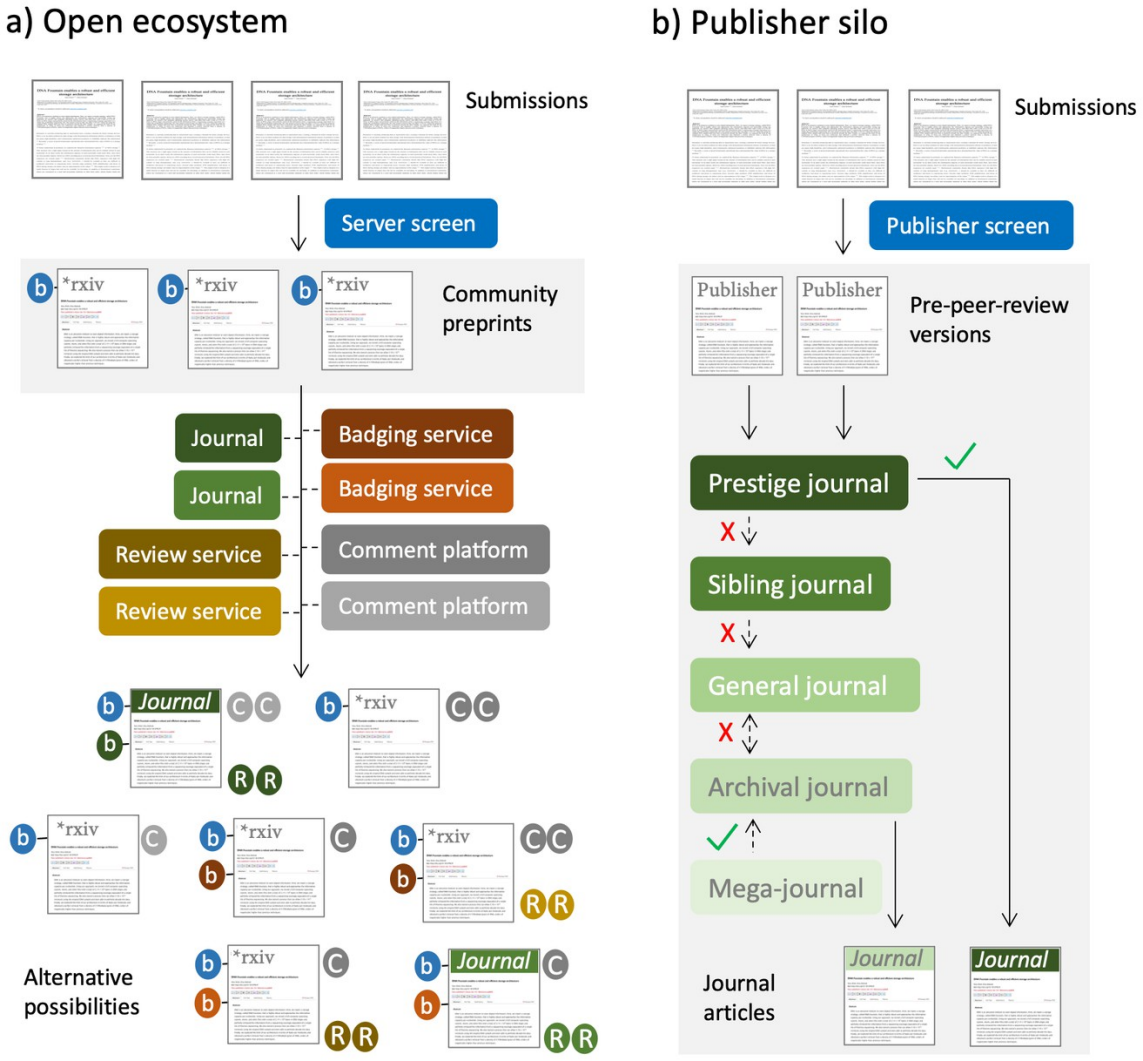
Contrasting visions of the future. (**a**) In an open ecosystem, dissemination by a preprint server (*rxiv) and evaluation are decoupled, and there are multiple alternative options for peer review and content verification. Content verification can be performed by various actors, resulting in the assignment of badges (b) that function as trust signals. Decoupled review (R) can be performed by journals or independent entities and coexists with unsolicited comments (C). (**b**) Commercial lock-in by contrast creates a walled garden where all these services are coordinated by a single publishing house and, once a paper is initially captured, it is retained and all that remains to be decided is the final destination within its hierarchy of journals following either a trickle-down or bubble-up process.

Best practices are just beginning to be established for preprints [44], and there is some way to go in standardizing how different versions of articles should be distinguished, cited, indexed, and linked. Nevertheless, it is now easy to envision a future in which the vast majority of papers are first disseminated as preprints and only afterwards undergo evaluation (Fig 5) and curation. For a very small number of findings that might present a danger to the public—for example, biosecurity risks or results that contradict critical public health guidance—alternate workflows involving preregistration or additional checks may be desirable [45]. This underscores the need for responsible screening of preprints before they are made public not simply unvetted immediate dissemination [20].

Biomedical scientists will need to adjust to this new norm, as will journalists and the wider community. There is an opportunity to educate lay readers about the nature of scientific enquiry, peer review, and how scientific consensus emerges as a consequence of multiple studies and self-correction. Preprints provide a timely focus on this against the background of a growing deluge of un-peer-reviewed, peer-reviewed, and allegedly peer-reviewed material. Again, the COVID-19 pandemic has been illustrative. It led to unprecedented public attention on preprints, with good journalists frequently noting the findings were “not yet peer reviewed.” Misinformation is obviously a concern but narratives that place preprints at the heart of it are frequently spurious [46], and articles that carry a false imprimatur of peer review and thus validity are arguably a bigger worry.

### Content verification in a decoupled system

The phrase “publish-review-curate” (PRC) is sometimes used to describe how science publishing should operate in a post-preprint world, and decoupled models for peer review are beginning to emerge (see below). However, it is important to remember there are forms of content verification one cannot expect peer reviewers or readers to perform. We must be careful not to overlook the need for these, particularly given the prevalence of image manipulation [47], the ease with which manuscripts can be generated computationally [48], and the emergence of paper mills fueled by a publish-or-perish culture [49]. Checks for plagiarism, fraud, image manipulation, adherence to relevant ethical and subject area norms, data/code availability, and other desirable scientific practices are important and have the potential to serve as key trust signals for the scientific community and the general public [50].

This is an area where improvement is needed, since many journals do little in the way of content verification and of those that do few are transparent about the checks they perform. A more granular and transparent approach would be significantly better than leaving readers to make assumptions based on journal brand. Assignment of “badges” to articles after passing specific content checks would provide more multidimensional signals for human readers, and embedding such badges in article metadata would be valuable for machine reading and indexing. The Open Researcher and Contributor ID (ORCID) scheme (https://orcid.org/) is an example. Although currently an author-disambiguation system, it is an important trust signal when confirmed by an author and/or attached to multiple articles in different venues, and one can envisage it evolving to become a system for identity verification. Both the Center for Open Science (COS) and the American Society for Cell Biology (ASCB) have developed badges for content that represent another step in this direction [51,52], while PLOS and other publishers have also indicated their intent to explore badging [53].

Content checks can take place at various stages in the life cycle of an article. Automated checks such as plagiarism screening can be performed by preprint servers prior to dissemination, and various manual checks are also desirable at this stage. medRxiv, for example, includes various ethics checks as part of its screening process for clinical submissions. Given the challenges preprint servers face in rapid processing of huge numbers of submissions, some more labor-intensive checks should be performed downstream by journals or third-party services. Many of the submission checklists operated by journals lend themselves to outputs of this nature. One possibility is that journals could assign badges to preprints as they conclude checks on submitted papers (i.e., before peer review is complete) so that this information is available earlier. Alternatively, third parties may perform these services. Initiatives like DataSeer (https://dataseer.ai/) and SciScore (https://www.sciscore.com/) are already exploring this territory. Either way, in the PRC parlance one can imagine a blend of VPRC and PVRC (where V denotes verification) in which content checks are performed at various points and badges are sequentially added to articles to give readers increased confidence (see Fig 5). A taxonomy of badging would need to be established for it to be useful, and clearly there would need to be some form of registration and verification of the services themselves. An important question is whether badges should be used solely to represent the kinds of binary/quantitative output from content checks or also encompass more subjective assessments by peer reviewers.

### Peer review possibilities

Decoupling of peer review from dissemination means we have an opportunity to rethink how peer review is performed, who should do it, when, and for which papers. In doing so, we should also ask who peer review is for: authors, specialist readers, general readers, the public, funders? The answers may differ depending on the paper and the subject.

It is sometimes argued that peer review should be abandoned entirely (see, for example, [54]) and so in a post-preprint world, readers should simply assess papers themselves. But we must be honest and admit many readers are not able to judge specialist content themselves. This is increasingly the case in multidisciplinary work. An oncologist, for example, may not be sufficiently knowledgeable to assess a crystal structure in a paper on a tumor suppressor but nevertheless want some assurance from an expert of its validity. Moreover, funders and employers effectively use peer review as an audit of their spending, and as work becomes of more general or clinical interest, the non-specialist audience grows. So there are stakeholders who never read papers themselves but nevertheless have an interest in seeing that the work is vetted. That said, we probably do not need to formally peer review all of the millions of papers written each year. Many findings are of interest only to a small number of experts perfectly qualified to evaluate the work themselves. The time and money spent organizing peer review of these papers is probably unnecessary, and some minimal set of content checks (see above) may be sufficient. For this approach to work, however, funders, universities, and other hiring institutions must recognize work that has not been formally reviewed as a legitimate research output. The Howard Hughes Medical Institute (HHMI) currently does so in its investigator evaluations [55], but many other funders insist on “peer-reviewed” publications, usually without actually defining what that means.

The prior availability of articles as preprints allows a spectrum of review activities to occur and accumulate—from informal commenting to more organized approaches (Fig 5a). A number of journal-independent peer review initiatives that complement or substitute for journal peer review are emerging across this spectrum, from bottom-up community initiatives such as PreReview to services like Peer Community In and Review Commons that more closely resemble the process performed by journals [56]. These approaches have the potential to add trust signals to papers that accumulate over time, particularly for papers that do not undergo formal journal review, and overlay journals may appear in this space (Box 2). They also represent an opportunity for self-organizing groups of academics to engage in peer review. An example is Biophysics Collab (https://www.sciencecolab.org/biophysics-colab), a collaboration of research scientists who review and curate preprints in biophysics. Creating tools for such groups to arrange peer review could help address some of the inequity and skewing among those who get to participate in the process [30]. It is important we ensure their activities are both transparent and discoverable. Tools like the bioRxiv dashboard (https://connect.biorxiv.org/news/2021/05/14/dashboard), Sciety (https://sciety.org/), and Early Evidence Base (https://eeb.embo.org) already aggregate reviews and commentary from a variety of sources, but ultimately it will be important that search engines and indexing services such as Crossref, Web of Science, and ideally PubMed incorporate these as well. Effective indexing of accumulating peer reviews would pave the way for a more open ecosystem in which a variety of different approaches to evaluation coexist (Fig 5a).

Decoupling also provides an opportunity to tailor peer review and further break it down. Peer review as currently performed is fairly uniform across different fields (i.e., 3 reviewers expected to provide unstructured written reports in 14 days). Perhaps, it should be done differently depending on the type of research. For example, should experimental and computational biology be reviewed differently, and might studies reporting new tools and techniques benefit from review processes more like road testing than traditional review? Similarly, preregistration, and therefore linking to peer reviews conducted before the work is performed, could be employed more broadly. While unlikely to be appropriate for a lot of exploratory research, this would help to avoid bias in reporting of studies for which a clear prior hypothesis can be registered.

Where different forms of review of the same paper are required, these could be separated. Statistical review at some journals currently involves dedicated reviewers, and this could take place on a different timescale with the outputs available earlier and appended as badges to preprints. We could do the same for other aspects of papers requiring specialist review. More generally, we should consider distinguishing “technical” review of methodology from “contextual” review of the findings. This would provide an opportunity to involve more ECRs in the process, particularly given that they tend to have more direct experience of new techniques than older principal investigators (PIs). ECRs in particular would also benefit from peer review being more broadly recognized as an academic contribution in its own right [57]. Tools like ORCID and Web of Science Reviewer Recognition Services (formerly called Publons) can help enable this, and it could further incentivize open/transparent peer review and consequently create additional trust signals associated with papers.

Perhaps the greatest inefficiency in the current publishing process is the successive, redundant reviewing of papers by different journals, in which the peer reviews for rejected papers remain hidden and represent millions of hours of lost scholarship. Trickle-down or bubble-up approaches that keep manuscripts within a publisher silo are one approach to combating this (Fig 5b). Cross-publisher approaches such as the Neuroscience Consortium in which peer reviews are passed between journals have been less successful [58], probably as a consequence of technical and cultural obstacles, but there is enthusiasm for portable peer review (see, for example, [59]). Public posting of reviews alongside preprints in an increasingly open system would help, but we must be mindful of authors wary of “metadata of failure” forever being associated with their papers. A significant advantage of reconceiving peer review as an accumulation of information is that it means potentially erroneous judgments can be superseded and allows later evaluations to occur, thus addressing one of the greatest weaknesses of the current approach: cementing a judgment made at a single point in time. This could be particularly important for research that is potentially actionable and/or of general interest. Given the prevalence of misinterpretation-based misinformation, the ability to add additional later reassessments and context could be very useful.

Normalizing such a process will be a cultural challenge. The PubPeer service provides the most obvious example of this and offers some useful lessons [60]. Undoubtedly a valuable service, PubPeer is sometimes viewed as controversial and authors frequently fail to respond to criticism there. As we consider how ongoing review, in particular corrections and retractions, should operate as part of a more decoupled approach, it will be important to define where authors’ obligations to respond begin and end, whose challenges warrant addressing, and who is the arbiter of this. Authors do not always respond well, and institutions have a history of delaying or failing to take responsibility when ethical issues are raised about research performed by their faculty.

### Curation and evaluation

Filtering of content has the potential to be much improved in a decoupled system that no longer relies so heavily on journal brand. Algorithmic recommendations based on text and citation analysis are already used widely. It’s a concern that a broader variety of tools are not routinely employed. Despite the appearance of numerous recommendation engines in recent years (Epistemic AI, Meta, Microsoft Academic, PubChase, PubCrawler, Semantic Scholar, etc.), many have been shuttered or failed to gain traction, and most biologists continue to rely on PubMed and Google Scholar. With the arrival of ChatGPT and other large language models (LLMs) blurring the lines between dialog and search, it will be interesting to see what role they play [61]. More choices would be welcome and avoid the biases of a single tool becoming baked into the literature graph.

Human curation will of course still play a role, be it via informal, trusted sources like colleagues and social networks or more organized tertiary material. Will this mean the return of the brand or the revenge of the community? Journal front sections (News & Views, Mini-Reviews, Perspectives, etc.) already play an important role highlighting work of potential interest. As journals cease to be venues where research itself first appears, providing context and analysis should become more important (much as newspapers are increasingly venues one looks to for analysis rather than places one first hears about events). Faculty Opinions (formerly Faculty of 1000) provides this service independently of journals [62], and the growing number of preprints in biology may make it more appealing since these are not already associated with a journal brand. PreLights is a journal-independent preprint-highlighting service that may serve as a model [63]. Scientific societies have a huge opportunity in this space given their standing as trusted sources of expertise and their membership reach. As ever, much of the discussion will revolve around business models; societies have tended to be extremely risk averse in the past.

Opinions differ on the desirability of quantitative indicators for articles, which can be used for both curation and evaluation. Goodhart’s Law applies, but there is a strong push to rank content. Academia is a prestige economy, and hypercompetition only fuels inflation within it. Citations are a widely used metric easily incorporated into search tools and evaluations. Other article-level metrics, such as downloads and social media-based measures of attention (e.g., altmetrics), now often appear alongside articles but there is little sign they are widely used. In both cases, numbers are hard to compare across disciplines, despite attempts to normalize for this [64], and attention is not always an indication of quality. There have been various calls for new ranking systems, including proposals akin to Yelp, Uber, eBay, or Amazon ratings. User ratings have their own issues though [65], which hardly make them a good model for academia. Plaudit is one such experiment. It has been incorporated by preprint servers on the Open Science Framework but has yet to see broader adoption [66]. Others have proposed vote- or reputation-based approaches like Reddit or Stack Exchange, but it’s hard to imagine active reader engagement on the scale needed, particularly from those whose opinions are desired, or a single system that operates across several million papers. And adding popularity as a further confounder to quality assessment is unlikely to help. Ultimately, evaluation is an issue institutions and funding agencies not publishers must address and avoid the pull of easy quality proxies. Recognizing that papers are merely narratives about research and just one of various research outputs is an important step in the right direction some funders and hiring institutions are taking.

One final aspect of curation to consider is whether there is a conflationary counterforce. An implicit assumption of the PRC model is that (peer) review and curation can be distinguished and occur in series. But if peer review is ongoing, the line between peer review and News & Views may blur as people experiment with evaluation. This could be important when opinions of the merits or validity of work differ among experts and/or later developments cast the work in a new light. In such cases, PRCR may be a more accurate depiction of events than PRC. We must be careful to avoid the wisdom of hindsight in judging individuals though, understanding that their logic, methods, and conclusions may have been perfectly reasonable given the state of knowledge at the time.

### Concluding remarks

Any vision for the future of science publishing should be centered on making new findings available as soon as possible to spur further research and speed up its translation to actionable tools and knowledge that benefits society. Peer review in its broadest sense should be a critical aspect of this process. In recent years, however, it has too often served as a mechanism for assigning quality proxies to articles, as academia has effectively outsourced evaluation of scientists to journals. We should instead focus efforts on ways to verify the content of articles and their authors and provide trust signals for readers. Decoupling peer review from dissemination will allow it to take on different forms, operated by different actors, among a variety of multidimensional indicators rather than sustaining a single quantitative or qualitative journal-based parameter. The oft-referenced “article of the future” we should strive for should thus be a constellation of linked web objects that include narrative (the article), data deposited in appropriate repositories, appropriately archived code and coding environments, verification badges, and any necessary protocols or preregistered research plans, amid a cloud of review and evaluation elements that accumulate over time.

To achieve this vision, we should strive for an evolving, open ecosystem (Fig 5a) rather than publisher silos that lock in content and assign immutable journal-based leading indicators early in their lifespan (Fig 5b). Achieving such a vision will require concerted efforts to achieve interoperability across infrastructure maintained by numerous different groups. It will also take buy-in and efforts from all stakeholders. Far too often have institutions and individuals lamented the state of scientific publishing while adopting counterproductive practices and failing to acknowledge that many of its undesirable characteristics emerged because of deeper issues in academia [26]. It is more than publishing that needs to be reformed.

## Abbreviations

APC: article-processing charge
ASCB: American Society for Cell Biology
COS: Center for Open Science
DOI: digital object identifier
DORA: Declaration on Research Assessment
ECR: early career researcher
HHMI: Howard Hughes Medical Institute
ICMJE: International Committee of Medical Journal Editors
LLM: large language model
OA: Open Access
ORCID: Open Researcher and Contributor ID
PI: principal investigator
PRC: publish-review-curate
TOP: Transparency and Openness Promotion
WW2: World War II
